# Modelling *Drosophila* motion vision pathways for decoding the direction of translating objects against cluttered moving backgrounds

**DOI:** 10.1007/s00422-020-00841-x

**Published:** 2020-07-04

**Authors:** Qinbing Fu, Shigang Yue

**Affiliations:** 1 Machine Life and Intelligence Research Centre, Guangzhou University, Guangzhou, China; 2 Computational Intelligence Lab/Lincoln Centre for Autonomous Systems, University of Lincoln, Lincoln, UK

**Keywords:** *Drosophila*, Motion vision, ON and OFF pathways, Direction selectivity, Visual system model, Foreground translation perception

## Abstract

Decoding the direction of translating objects in front of cluttered moving backgrounds, accurately and efficiently, is still a challenging problem. In nature, lightweight and low-powered flying insects apply motion vision to detect a moving target in highly variable environments during flight, which are excellent paradigms to learn motion perception strategies. This paper investigates the fruit fly *Drosophila* motion vision pathways and presents computational modelling based on cutting-edge physiological researches. The proposed visual system model features bio-plausible ON and OFF pathways, wide-field horizontal-sensitive (HS) and vertical-sensitive (VS) systems. The main contributions of this research are on two aspects: (1) the proposed model articulates the forming of both direction-selective and direction-opponent responses, revealed as principal features of motion perception neural circuits, in a feed-forward manner; (2) it also shows robust direction selectivity to translating objects in front of cluttered moving backgrounds, via the modelling of spatiotemporal dynamics including combination of motion pre-filtering mechanisms and ensembles of local correlators inside both the ON and OFF pathways, which works effectively to suppress irrelevant background motion or distractors, and to improve the dynamic response. Accordingly, the direction of translating objects is decoded as global responses of both the HS and VS systems with positive or negative output indicating preferred-direction or null-direction translation. The experiments have verified the effectiveness of the proposed neural system model, and demonstrated its responsive preference to faster-moving, higher-contrast and larger-size targets embedded in cluttered moving backgrounds.

## Introduction

Intelligence is one of the amazing products through millions of years of evolutionary development, with which the features of biological visual systems have been gradually learnt and acknowledged as powerful model systems towards building robust artificial visual systems. In nature, for the vast majority of animal species, a critically important feature of visual systems is the perception and analysis of motion that serves a wealth of daily tasks for animals (Borst and Euler 2011; Borst and Helmstaedter 2015). Seeing the motion and direction in which a chased prey, a striking predator, or a mating partner is moving, is of particular importance for their survival.

Direction-selective neurons, with responsive preference to specific directional visual motion, have been identified in flying insects, like locusts (Rind 1990) and flies (Borst and Euler 2011). Each group of the direction selective neurons responds selectively to a specific optic flow (OF)-field representing the spatial distribution of motion vectors on the field of vision. Accordingly, the visual motion cues as feedback signals provided by such neurons are applied for ego-motion control of flying insects.

Recent decades have witnessed much progress on unravelling the underlying neurons, pathways and mechanisms of insects’ motion vision systems (Fu et al. 2019b). Notably, the fruit fly *Drosophila* has been disseminated as a prominent paradigm to study motion perception strategies (Riehle and Franceschini 1984; Franceschini et al. 1989; Borst and Euler 2011; Borst and Helmstaedter 2015; Borst et al. 2010; Borst 2014). More specifically, direction-selective (DS) and direction-opponent (DO) responses represented by the *Drosophila* motion vision pathways have been identified as two essential features in the neural circuits (Mauss et al. 2015; Haag et al. 2016; Badwan et al. 2019). The former indicates that neurons respond differently to stimuli moving in different directions, that is, the directional motion yielding the largest response is termed the preferred direction (PD); the latter denotes that neurons are also inhibited by stimuli in the opposite direction, i.e. the null (or non-preferred) direction (ND). How to realise such diverse direction selectivity in motion-sensitive visual systems is thus attractive to not only biologists but also computational modellers for addressing real-world motion detection problems.

Although some biological and computational models have demonstrated the DS and DO responses resembling the neural circuits to decode the direction of translational OF, those models are faced with the following challenges:

1. The biological models focus on explaining the forming of DS and DO responses on neuronal or behavioural level, which have been tested by merely simple synthetic stimuli, e.g. sinusoidal gratings and the like (Joesch et al. 2010, 2013; Maisak et al. 2013; Eichner et al. 2011; Clark et al. 2011; Haag et al. 2016; Gabbiani and Jones 2011). Flying insects nevertheless can detect and track a moving target in front of more cluttered backgrounds mixed with irrelevant motion or distractors. Are these models able to reproduce the similar DS and DO responses when dealing with the highly variable statistics of natural environments? This is yet lack of investigation.
2. It is still a challenging problem for artificial visual systems to accurately decode the direction of foreground translating objects, by extracting merely meaningful motion cues embedded in a cluttered moving background. The vast majority of bio-inspired models are efficient for motion perception, but deficient in effective mechanisms to deal with highly variable backgrounds. In addition, the requirements of both energy-efficient and real-time visual processing exclude many segmentation or learning-based methods.
3. Most bio-inspired motion detection models derive from a classic theory of Hassenstein–Reichardt correlation (HRC, or referred as ‘Reichardt detectors’) (Hassenstein and Reichardt 1956; Borst and Egelhaaf 1989). The HRC-based models are sensitive to the temporal frequency of visual stimuli across the view rather than the true velocity. Accordingly, a pronounced shortcoming of such methods is the dynamic response in speed tuning of translational OF perception (Frye 2015; Zanker et al. 1999).

In this article, according to the latest physiological researches and our preliminary studies on the *Drosophila* motion vision systems (Fu and Yue 2017a, b; Fu et al. 2018), we present a thorough modelling study to mimic the visual processing in *Drosophila* ON and OFF motion vision pathways through multiple layers, from initial photoreceptors to internal lobula plate tangential cells (LPTCs), in a computational manner. Differently to previous related methods, the emphasis herein is laid behind the OF level. More specifically, we highlight the modelling of spatiotemporal dynamics in the proposed neural system model including 1) the combination of spatial and temporal motion pre-filtering mechanisms prior to generating the DS and DO responses, 2) the ensembles (or multi-connected) of local ON–ON and OFF–OFF motion correlators inside the ON and OFF pathways in horizontal and vertical directions. The former works effectively to suppress irrelevant background motion flows or distractors to a large extent, and to achieve edge selectivity revealed in motion detection neural circuits. The latter can enhance the dynamic response to translating objects in front of a cluttered moving background, and alleviate the impact by temporal frequency of visual stimuli. Accordingly, two wide-field systems, i.e. the horizontal-sensitive (HS) and the vertical-sensitive (VS) systems, integrate the LPTCs’ responses to decode the principal direction of foreground translating objects against cluttered moving backgrounds.

The rest of this paper is structured as follows. Section 2 reviews the related works. Section 3 presents the formulation of the proposed visual system model. Section 4 describes the experimental setting. Section 5 illustrates the results. Section 6 presents further discussions. Section 7 concludes this paper.

## Literature review

Within this section, we concisely review the related works in the areas of (1) a few categories of motion-sensitive neural models inspired by flying insects, (2) physiological research on the *Drosophila* motion vision pathways, (3) different combinations of the EMD in the ON and OFF channels. The nomenclature is given in Table 1.

**Table 1:** Nomenclature in this paper

Acronym and full name			
EMD	Elementary motion detector	HRC	Hassenstein–Reichardt correlation
DS	Direction-selective	DO	Direction-opponent
HS	Horizontal-sensitive	VS	Vertical-sensitive
PD	Preferred direction	ND	Null (or non-preferred) direction
OF	Optic flow	LMC	Lamina monopolar cell
vDoG	Variant of Difference of Gaussians	FDSR	Fast-depolarising-slow-repolarising
LPTC	Lobula plate tangential cell	LPi	Lobula plate-intrinsic
LGMD	Lobula giant movement detector	STMD	Small target movement detector

### Motion-sensitive neural models

Flying insects have tiny brains, but compact visual systems for decoding diverse motion features varying in directions and sizes. Some identified neurons and corresponding circuits have been investigated as robust motion-sensitive neural models, as reviewed in Fu et al. (2019b).

In the locust’s visual brains, two lobula giant movement detectors (LGMDs), i.e. the LGMD-1 and the LGMD-2, have been modelled as quick and robust looming detectors specialising in collision perception (Fu et al. 2016, 2017, 2018b, 2019a). The LGMD models respond selectively to movements in depth, with the most powerful response to objects that signal frontal collision threats. A good number of models have been applied for collision detection against various scenarios including ground vehicles, mobile robots and unmanned aerial vehicles (Fu et al. 2018a, 2019b).

Inspired by the flies and bees, a considerable number of OF-based collision sensing visual systems mimics the functions of bilateral compound eyes, at ommatidium level. More specifically, there are several categories of methods to realise such signal processing. The HRC theory originates the elementary motion detector (EMD)-based models correlating two signals in space, by multiplication with one delayed (Borst and Egelhaaf 1989); such a method is effective to enhance the PD motion. Another famous mechanism is called the “Barlow–Levick” model by nonlinearly suppressing the ND motion (Barlow and Levick 1965), which is recently collaborated with the HRC mechanism in constructing fly motion detectors (Strother et al. 2017; Haag et al. 2016). In addition, Franceschini proposed a velocity-tuned method depending on the ratio between the photoreceptor angles in space and the time delay for each pairwise contrast detection photoreceptor (Franceschini et al. 1989, 1992); subsequently, it has been called the “time-of-travel” scheme (Moeckel and Liu 2007; Vanhoutte et al. 2017). As a variation of the EMD, a few methods were proposed to decode or estimate the angular velocity accounting for various flight behaviours of bees (Brinkworth and O’Carroll 2009; Cope et al. 2016; Wang et al. 2019a, b). Benefiting from the computational efficiency and robustness, many OF-based methods have been applied for near-range navigation of flying robots and micro-aerial vehicles, as reviewed in (Franceschini 2014; Serres and Ruffier 2017).

With distinct size selectivity, the small target movement detectors (STMDs) in flying insects, like the dragonflies, respond selectively to moving objects of very small size (subtended an angle of less than 10$^\circ$) (Fu et al. 2019b). Wiederman proposed seminal works to detect small dark object motions embedded in natural scenes, via correlating ON and OFF channels in motion detection circuits (Wiederman et al. 2008, 2013). They also combined its functionality with the EMD structure to implement the direction selectivity (Wiederman and O’Carroll 2013). Recently, the STMD models have been successfully implemented in a ground robot to track small targets in natural backgrounds (Bagheri et al. 2017), and in on-line system of an airborne vehicle for small-field object detection and avoidance (Escobar et al. 2019).

### Physiological research on the fly motion vision pathways

**Fig. 1:**
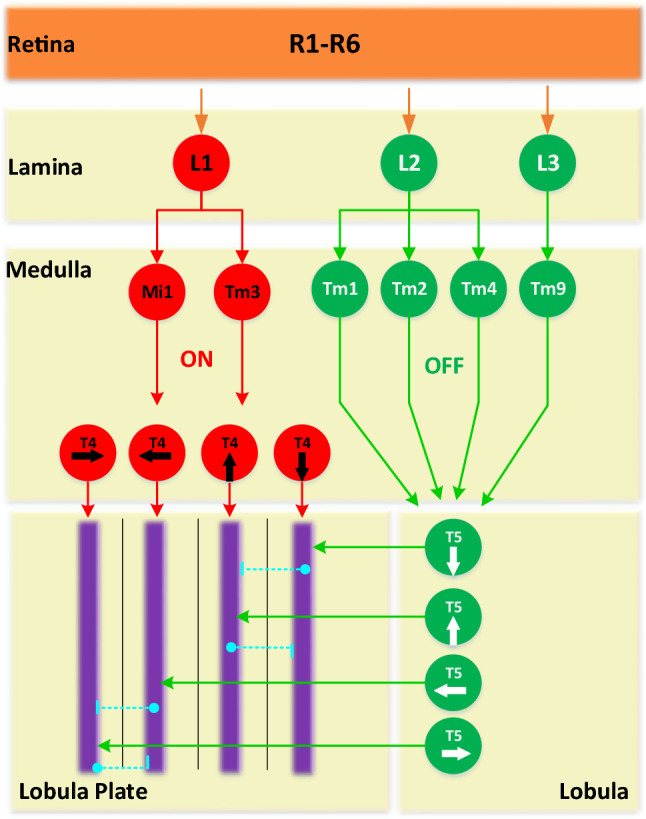
Schematic diagram of the *Drosophila* ON and OFF motion vision pathways with five neuropile layers: the first retina layer R1–R6 neurons convey motion information to lamina monopolar cells (LMCs, i.e. L1, L2, L3); the signals are then split into parallel ON and OFF channels denoted by different coloured neurons and pathways; the directionally selective signals are carried via T4 and T5 cells to four sub-layers of the lobula plate, where T4 and T5 cells with the same PD signals converge on the same dendrites of the tangential cells; the inhibition is conveyed via lobula plate-intrinsic (LPi) interneurons (dashed lines) between stratified neighbouring layers in the lobula plate

Our proposed model is based on an important physiological theory that motion information is processed in parallel ON and OFF pathways (Borst et al. 2020). As illustrated in Fig. 1, we can summarise the following steps of the *Drosophila*’s preliminary visual processing:

1. The motion perception starts from the retina layer with photoreceptors (R1–R6) which conveys received brightness to LMCs in the lamina layer.
2. The LMCs encode motion by luminance increments (ON) and decrements (OFF). The motion information is separated into parallel channels: the L1 with its downstream Mi1 and Tm3 interneurons in the medulla layer convey onset or light-on response to succeeding T4 neurons in the medulla layer, while the L2, L3 with their downstream Tm1, Tm2, Tm4 and Tm9 interneurons relay offset or light-off response to subsequent T5 neurons in the lobula layer (Rister et al. 2007; Haag et al. 2016; Strother et al. 2014; Fisher et al. 2015).
3. The DS responses of ON and OFF contrasts are produced by the T4 and T5 cells in a feed-forward manner, respectively. The selectivity to four cardinal directions is well separated in different groups of T4 and T5 neurons (Maisak et al. 2013).
4. The LPTCs in four stratified sub-layers of the lobula plate integrate the T4 and T5 signals, where the same DS responses converge on the same sub-layer. Meanwhile, the LPi interneurons convey inhibition to adjacent sub-layers through sign-inverting interactions, thus forming the DO responses (Mauss et al. 2015). Finally, two directionally selective systems, i.e. the HS and VS systems pool the responses from LPTCs towards sensorimotor control (Joesch et al. 2010).

### EMD in the ON and OFF Channels

**Fig. 2:**
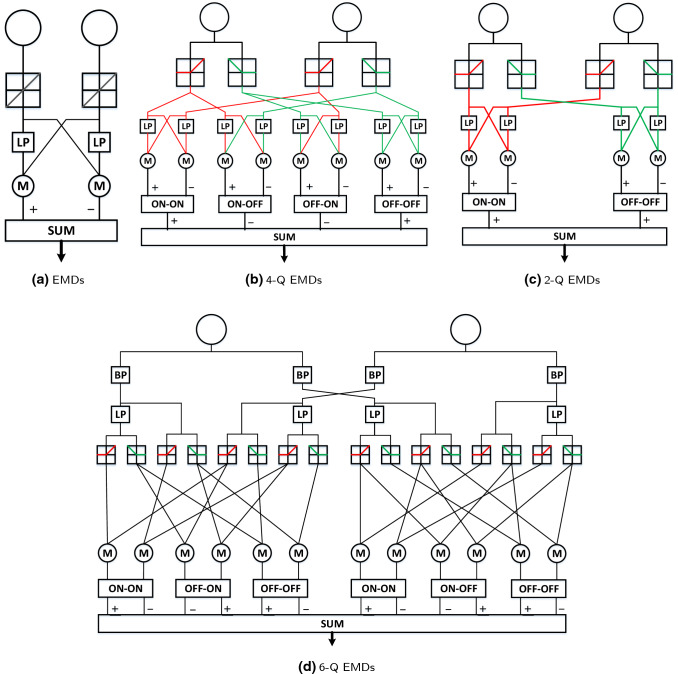
Different combinations of the EMD in the ON and OFF channels: LP, BP and M components indicate the low-pass filtering, the band-pass filtering and the multiplication processes

Based on the EMD, there are several theories representing different combination forms to encode the spatiotemporal signal flows in the ON and OFF channels (Joesch et al. 2013), as shown in Fig. 2. Importantly, since the signals are already directionally selective before collectively arriving at the stratified LPTCs (Maisak et al. 2013; Badwan et al. 2019), all these models could well explain the neural computation inside the ON and OFF channels.

More concretely, in the former EMD models, e.g. (Iida and Lambrinos 2000; Zanker and Zanker 2005; Zanker et al. 1999), visual information is processed in a single pathway with the basic format between every pairwise photoreceptors (Fig. 2a). After the identification of ON and OFF channels, the motion information is split into different places, however can further interact even with the opposite polarity signal flows. Different combinations have been investigated through either the electro-physiological recordings from the LPTCs (Eichner et al. 2011) or the behavioural experiments (Clark et al. 2011). The 4-quadrant (4-Q) detectors model with communications between both the same and the opposite polarity signals (see Fig. 2b) is fully equivalent to the full-HRC, namely the EMD model in Fig. 2a. The second important model is the 2-Q structure in Fig. 2c, which processes input combinations of only the same-sign signals, i.e. ON–ON and OFF–OFF contrast. In addition to that, the 6-Q model has a more complex structure (see Fig. 2d), which argues that either the ON/OFF channel conveys motion information with both positive (onset) and negative (offset) contrast changes. Our proposed method leverages them with the 2-Q model’s simpler computational structure as well as the 6-Q model’s edge selectivity prior to the ON and OFF channels.

## Formulation of the proposed model

**Fig. 3:**
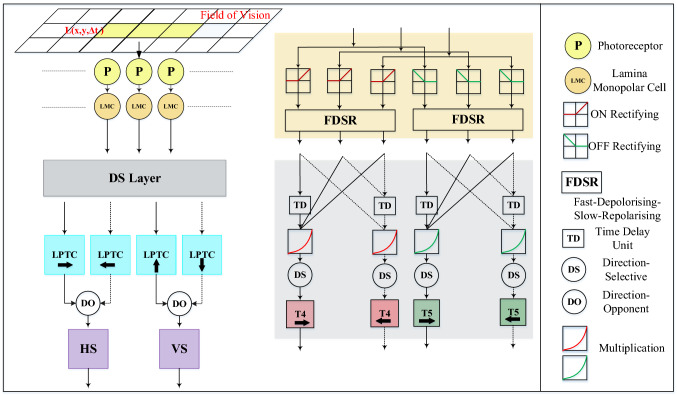
Schematic illustration of the proposed model consisting of the ON and OFF motion pathways throughout several computational layers mimicking the *Drosophila* physiology in Fig. 1. The DS layer exemplifies the processing of each interneuron interacting with two horizontal neighbour cells in the medulla and lobula layers, where the dashed lines with respect to the solid ones indicate the generation and transmission of opposing DS motion

**Fig. 4:**
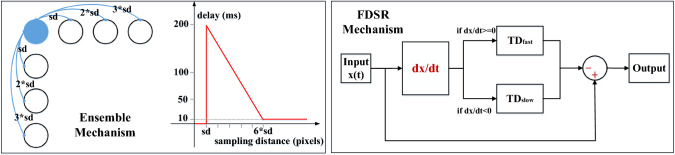
Illustrations of the proposed mechanisms of spatiotemporal dynamics inside the ON and OFF pathways. The left sub-figure exemplifies the ensembles of local motion detectors in two directions, where the latency is dynamic depending on the sampling distance (sd) between each pair-wise correlators. The right sub-figure depicts the temporal FDSR mechanism

Within this section, we present the formulation of the proposed visual system model. Figure 3 depicts the schematic of model structure. Generally speaking, for mimicking the *Drosophila* physiology in Fig. 1, the model consists of mainly five computational neuropile layers with the HS and VS systems. The forming of DS and DO responses in the proposed model resembles the revealed *Drosophila* visual processing in a feed-forward manner (Badwan et al. 2019). Compared to previous related methods, we highlight the following mechanisms:

1. The model combines bio-plausible spatiotemporal pre-filtering methods to remove redundant background motion to a large extent, and achieve edge selectivity, which include firstly a variant of ‘Difference of Gaussians’ (vDoG) mechanism with ON and OFF contrast selectivity, spatially, and then a fast-depolarising–slow-repolarising (FDSR) mechanism, temporally (see Fig. 4).
2. To improve the dynamic response and alleviate the impact by temporal frequency of visual stimuli, we propose a novel structure representing ensembles of motion correlators for each interneuron inside the ON and OFF pathways to produce the DS responses in horizontal and vertical directions, i.e. multi-connected and same-polarity cells possess dynamic latency corresponding to the sampling distance between each pairwise detectors (see Fig. 4).
3. The HS and VS systems integrate the local DS responses from stratified LPTCs with inhibitions from adjacent LPi interneurons representing the DO responses to form global membrane potential. Accordingly, the PD or ND translating motion is indicated by the positive or negative membrane potential of both systems.

### Computational retina layer

In the first retina layer, there are photoreceptors (see P in Fig. 3) that capture single-channel luminance (green-channel or grey-scale in our case), at ommatidia (grouped local optical units) or local pixel level from images, with respect to time. Let $L(x,y,t) \in \mathbb {R}^3$ denote the input image streams, where *x*, *y* and *t* are the spatial and temporal positions. The calculation of motion signals is as follows:

1 $$\begin{aligned} P(x,y,t) = L(x,y,t) - L(x,y,t-1) + \sum _{i=1}^{n_p}a_i \cdot P(x,y,t-i). \end{aligned}$$

The change of brightness could continue and decay for a short while of $n_p$ number of frames. The decay coefficient $a_i$ is computed by

2 $$\begin{aligned} a_i = \left( 1 + e^{i}\right) ^{-1}. \end{aligned}$$

### Computational lamina layer

As illustrated in Fig. 1 and 3, in the second lamina layer, there are LMCs that split motion signals from the retina layer into parallel ON and OFF channels encoding light-on (onset) and light-off (offset) responses, respectively. For enhancing the motion edge selectivity and maximising the transmission of useful information from visually cluttered environments, we propose a bio-plausible spatial mechanism, named the “vDoG”, simulating the functions of LMCs. Compared to the traditional DoG mechanism, it also demonstrates the ON and OFF contrast selectivity to fit with the following processing in the ON and OFF channels. The vDoG depicts a centre-surrounding antagonism with centre-positive and surrounding-negative Gaussians representing excitatory and inhibitory fields in space. That is,

3 $$\begin{aligned} P_e(x,y,t)= & {} \sum _{u=-2}^{2}\sum _{v=-2}^{2} P(x-u,y-v,t) \cdot G_{\sigma _e}(u,v),\nonumber \\ P_i(x,y,t)= & {} \sum _{u=-4}^{4}\sum _{v=-4}^{4} P(x-u,y-v,t) \cdot G_{\sigma _i}(u,v), \end{aligned}$$

4 $$\begin{aligned} G_{\sigma _e}(u,v)= & {} \frac{1}{2\pi \sigma _e^2} \text {exp}\left( -\frac{u^2 + v^2}{2 \sigma _e^2} \right) ,\nonumber \\ G_{\sigma _i}(u,v)= & {} \frac{1}{2\pi \sigma _i^2} \text {exp}\left( -\frac{u^2 + v^2}{2 \sigma _i^2} \right) , \end{aligned}$$

where $\sigma _e$ and $\sigma _i$ indicate the excitatory and inhibitory standard deviations. The outer convolution kernel *G* is with twice the radius of the inner one. Accordingly, the broader inhibitory Gaussian is subtracted from the narrower excitatory one with the polarity selectivity. That is,

5 $$\begin{aligned}&LA(x,y,t)\nonumber \\&\quad = \left\{ \begin{aligned} |P_{e}(x,y,t) - P_{i}(x,y,t)|,&\text {if}\ P_{e}(x,y,t) \ge 0&\ P_{i}(x,y,t) \ge 0\\ -|P_{e}(x,y,t) - P_{i}(x,y,t)|,&\text {if}\ P_{e}(x,y,t)< 0&\ P_{i}(x,y,t) < 0 \end{aligned} \right. . \nonumber \\ \end{aligned}$$

After that, there are ON and OFF half-wave rectifying mechanisms splitting motion information into two parallel pathways, via filtering out negative and positive inputs for the ON and OFF pathways, respectively (see Fig. 3). In addition to that, the negative inputs to the OFF pathway are sign-inverted. The calculations are expressed as follows:

6 $$\begin{aligned} L1(x,y,t)= & {} [LA(x,y,t)]^{+}, \nonumber \\ L2(x,y,t)= & {} -[LA(x,y,t)]^{-}. \end{aligned}$$

$[x]^{+}$ and $[x]^{-}$ denote $\text {max}(0,x)$ and $\text {min}(x,0)$, respectively. *L*1 and *L*2 indicate the LMCs in the lamina layer, i.e. L1 in the ON channels, L2 and L3 in the OFF channels (see Fig. 1).

For each interneuron in the lamina layer, an ‘adaptation state’ is formed by the bio-plausible FDSR mechanism which matches the neural characteristic of ‘fast onset and slow decay’ phenomena. As depicted in Fig. 4, we first check the derivative of inputs from the ON and OFF channels along the time *t*. As digital signals do not have continuous derivatives, we compare neuronal responses between every two successive frames to get the change:

7 $$\begin{aligned} \varDelta L1(x,y)= & {} L1(x,y,t) - L1(x,y,t-1),\nonumber \\ \varDelta L2(x,y)= & {} L2(x,y,t) - L2(x,y,t-1). \end{aligned}$$

Subsequently, the input signals from the ON and OFF channels are delayed with two different latency constants $\tau _1, \tau _2$ in milliseconds, and $\tau _1<\tau _2$ representing the ‘fast depolarising’ with non-negative change and the ‘slow repolarising’ with negative change, respectively. That is,

8 $$\begin{aligned} \begin{aligned}&\hat{L1}(x,y,t)\\&\quad = \left\{ \begin{aligned}&\alpha _1 L1(x,y,t)+(1-\alpha _1) L1(x,y,t-1),\ \text {if}\ \varDelta L1(x,y) \ge 0,\\&\alpha _2 L1(x,y,t)+(1-\alpha _2) L1(x,y,t-1),\ \text {if}\ \varDelta L1(x,y)< 0. \end{aligned} \right. \\&\hat{L2}(x,y,t)\\&\quad = \left\{ \begin{aligned}&\alpha _1 L2(x,y,t)+(1-\alpha _1) L2(x,y,t-1),\ \text {if}\ \varDelta L2(x,y) \ge 0,\\&\alpha _2 L2(x,y,t)+(1-\alpha _2) L2(x,y,t-1),\ \text {if}\ \varDelta L2(x,y) < 0. \end{aligned} \right. \end{aligned} \end{aligned}$$

9 $$\begin{aligned} \alpha _1 = \tau _i / (\tau _1 + \tau _i),\ \alpha _2 = \tau _i / (\tau _2 + \tau _i). \end{aligned}$$

$\tau _i$ is the discrete time interval in milliseconds, between frames. Notably, in the FDSR mechanism, the delayed signal is subtracted from the original one (see Fig. 4) as

10 $$\begin{aligned} M1(x,y,t)= & {} L1(x,y,t) - \hat{L1}(x,y,t), \nonumber \\ M2(x,y,t)= & {} L2(x,y,t) - \hat{L2}(x,y,t). \end{aligned}$$

*M*1 and *M*2 denote interneurons in the medulla layer including Mi1, Tm3 in the ON channels, and Tm1, Tm2, Tm4, Tm9 in the OFF channels (see Fig. 1). Such a temporal mechanism contributes significantly to filter out irrelevant background OF and visual flickers, like the windblown vegetation in natural environments.

### Computational medulla and lobula layers

Next, both the medulla and lobula layers constitute the DS layer in Fig. 3, in order to generate the specific DS responses to four cardinal directions, where those interneurons interact with each other, nonlinearly (Maisak et al. 2013). Importantly, like the genuine T4 and T5 neurons in several individual groups sensitive to different directions (see Fig. 1), the proposed model demonstrates the same directional tuning. As mentioned above, for each local cell in the DS layer, we propose the ensemble mechanism (see Fig. 4) connecting same polarity motion detectors, in space, with ON–ON contrast correlation in the ON pathway and OFF–OFF contrast correlation in the OFF pathway, separately. Each pair-wise connection is featured by the aforementioned 2-Q correlation structure (see Fig. 2c). In addition, as illustrated in Fig. 4, the delay is dynamic, varying with respect to the sampling distance between every pair-wise detectors. More precisely, the combination with smaller distance has larger latency, which decreases as the space increases. In our preliminary modelling and bio-robotic researches (Fu and Yue 2017b; Fu et al. 2018), such a multi-connected structure has demonstrated the improved dynamic response in speed tuning of translational OF perception, when challenged by a range of angular velocities. The computations for producing the DS responses of the T4 neurons in the medulla layer are defined as follows:

11 $$\begin{aligned} \begin{aligned}&T4_{r}(x,y,t) = \sum _{i=sd}^{sd \cdot n_c}\hat{M1}(x,y,t) \cdot M1(x+i,y,t),\\&T4_{l}(x,y,t) = \sum _{i=sd}^{sd \cdot n_c}\hat{M1}(x+i,y,t) \cdot M1(x,y,t),\\&T4_{d}(x,y,t) = \sum _{i=sd}^{sd \cdot n_c}\hat{M1}(x,y,t) \cdot M1(x,y+i,t),\\&T4_{u}(x,y,t) = \sum _{i=sd}^{sd \cdot n_c}\hat{M1}(x,y+i,t) \cdot M1(x,y,t). \end{aligned} \end{aligned}$$

$\{r, l, d, u\}$ indicate the DS responses on four cardinal directions: rightward, leftward, downward and upward. $n_c$ and *sd* stand for the number of correlated neighbouring cells for each original cell, and the sampling distance between each pair-wise combination, respectively. $\hat{M1}$ denotes the delayed signal, calculated by

12 $$\begin{aligned} \begin{aligned} \hat{M1}(x,y,t) =&\alpha _3 M1(x,y,t) + (1-\alpha _3) M1(x,y,t-1),\\&\alpha _3 = \tau _i / (\tau _i + \tau _s). \end{aligned} \end{aligned}$$

$\tau _s$ indicates the proposed dynamic time delay, as exemplified in Fig. 4. Similarly for the T5 neurons in the lobula layer, the forming of DS responses along four cardinal directions is expressed as follows:

13 $$\begin{aligned} \begin{aligned}&T5_{r}(x,y,t) = \sum _{i=sd}^{sd \cdot n_c}\hat{M2}(x,y,t) \cdot M2(x+i,y,t),\\&T5_{l}(x,y,t) = \sum _{i=sd}^{sd \cdot n_c}\hat{M2}(x+i,y,t) \cdot M2(x,y,t),\\&T5_{d}(x,y,t) = \sum _{i=sd}^{sd \cdot n_c}\hat{M2}(x,y,t) \cdot M2(x,y+i,t),\\&T5_{u}(x,y,t) = \sum _{i=sd}^{sd \cdot n_c}\hat{M2}(x,y+i,t) \cdot M2(x,y,t). \end{aligned} \end{aligned}$$

The delay computation of $\hat{M2}$ conforms to Eq. 12, which is not restated here.

Importantly, a latest biological research has revealed that the distinct DS responses are all generated in a feed-forward manner when arriving the T4 and T5 neurons, each group of which demonstrates the specific direction selectivity (Badwan et al. 2019). As introduced in Sect. 2, though the different mechanisms forming such DS responses, with either PD motion enhancement or ND motion suppression, are still in debate, the proposed visual system model reconciles well the generation of DS responses with feed-forward signal processing.

### Computational lobula plate layer

After that, as illustrated in Fig. 1, the LPTCs in four stratified sub-layers integrate the DS responses from different groups of T4 and T5 neurons each with specific PD motion tuning, where the same DS responses converge at an identical sub-layer of the lobula plate. That is,

14 $$\begin{aligned} \begin{aligned}&LP_r(t) = \sum _{x=1}^{C}\sum _{y=1}^{R}T4_{r}(x,y,t) + T5_{r}(x,y,t),\\&LP_l(t) = \sum _{x=1}^{C}\sum _{y=1}^{R}T4_{l}(x,y,t) + T5_{l}(x,y,t),\\&LP_d(t) = \sum _{x=1}^{C}\sum _{y=1}^{R}T4_{d}(x,y,t) + T5_{d}(x,y,t),\\&LP_u(t) = \sum _{x=1}^{C}\sum _{y=1}^{R}T4_{u}(x,y,t) + T5_{u}(x,y,t). \end{aligned} \end{aligned}$$

*C* and *R* indicate columns and rows of the two-dimensional visual field. In addition to that, the proposed DO responses by opposing motions are generated via a sign-inverting operation representing the functionality of LPi interneurons inhibiting the LPTCs in neighbouring sub-layer (see Figs. 1 and 3), which are pooled by the HS and VS systems as the following:

15 $$\begin{aligned} HS(t) = LP_r(t) - LP_l(t),\ VS(t) = LP_d(t) - LP_u(t). \end{aligned}$$

With regard to the nonlinear and symmetric mapping in each combination of local ON–ON or OFF–OFF motion correlators inside the ON and OFF pathways, the model response is tuned to be positive by the PD (rightward and downward) motions, while negative by the ND (leftward and upward) motions.

Finally, the global membrane potential of either the HS/VS system is activated by a sigmoid function. Let the *HS*(*t*) or *VS*(*t*) be *x*, the function is defined as

16 $$\begin{aligned} f(x) = 2 \cdot {\text {sgn}}(x) \cdot ((1 + e^{-|x| \cdot (C \cdot R \cdot k)^{-1}})^{-1} - 0.5), \end{aligned}$$

where *k* is a scale coefficient. Accordingly, the output of proposed model is regulated within [0, 1) for the positive input, and $(-1,0]$ for the negative input.

### Setting model parameters

**Table 2:** Setting parameters of the proposed visual system model

Parameter	Description	Value
$n_p$	Number of persistent frames	$0\sim 2$
$\tau _i$	Time interval constant between frames	1000/30 ms
$\tau _1$	Fast depolarising time constant	1 ms
$\tau _2$	Slow repolarising time constant	100 ms
*sd*	Sampling distance between each combination	4
$\{\sigma _e,\sigma _i\}$	Standard deviations in vDoG mechanism	$\{2,4\}$
$n_c$	Number of correlating cells	4
$\tau _s$	Dynamic delay in ensembles of correlators	$10\sim 200$ ms
$\{C,R\}$	Columns and rows of the visual field	adaptable
*k*	Coefficient in sigmoid function	0.01

The parameters of the proposed model are given in Table 2. All of them are decided, empirically, with considerations of the functionality of the *Drosophila* visual system, and based on our previous modelling and experimenting experience in Fu and Yue (2017a, (2017b), Fu et al. (2018). In particular, the parameters of the vDoG mechanism correspond to the sampling distance between local motion correlators ($sd=\sigma _i$). It is also worthwhile pointing out that since each local cell inside the ON and OFF channels correlates with multiple neighbouring cells in horizontal and vertical directions, increasing the number of correlating cells ($n_c$) could further improve the dynamic response to translating stimuli, at the cost though of more computational consumption.

## Experimental setting

In this section, we introduce the experimental settings. Generally speaking, all the experiments can be categorised into two types of tests. In the first type of tests, we aim at demonstrating the robust DS and DO responses, as the basic characteristic of the proposed neural system model, challenged by visual stimuli against various backgrounds, from simple to complex. More specifically, the visual inputs are with $320 \times 180$ pixels, at 30 frames per second (fps) for the clean and real-world scenarios. After that, more systematic experiments are carried on with two cluttered moving backgrounds. We simulate a bar with $25 \times 120$ pixels in size, at three certain grey levels (white, moderate, dark), translating rightward at three individual angular velocities of 9, 18, 27 degrees per second (degrees/s), in front of a cluttered moving background. Note that both the two backgrounds shift in an opposite direction (leftward) relative to the foreground translating targets, at a range of velocities ($-5, -10, -20, -30, -40 \,^\circ /\mathrm{s}$), respectively. The visual inputs are with $700 \times 180$ pixels, at 30 fps.

**Fig. 5:**
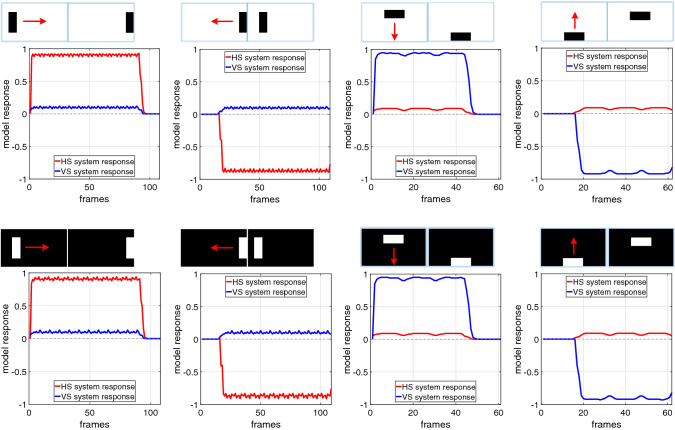
Proposed model responses challenged by dark and white objects translating constantly in four cardinal directions, against clean backgrounds

**Fig. 6:**
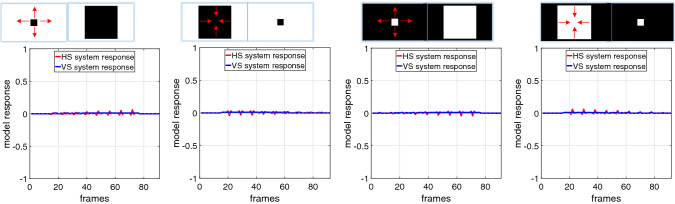
Proposed model responses by dark and white objects approaching and receding, against clean backgrounds

In the second type of tests, we look deeper into the properties of both model and stimuli considering the effects of proposed spatiotemporal dynamics on decoding the direction of translating objects in front of cluttered moving backgrounds. We compare the performance of models with and without the investigated mechanisms including the coordination of motion pre-filtering vDoG and FDSR, and the parameter $n_c$ (number of correlating detectors) in the ensembles of local ON–ON/OFF–OFF motion correlators. Moreover, we also give insight into the model performance on perception of different sized targets ($10 \times 10$, $25 \times 25$, $50 \times 50$ and $100 \times 100$ pixels), and also against very high-speed moving backgrounds ($-50, -100, -200 \,^\circ /\mathrm{s}$). The main objectives are 1) demonstrating the significance of proposed new structures and mechanisms for detecting the foreground translating objects and reproducing the DS/DO response; 2) revealing the model’s responsive preferences.

**Fig. 7:**
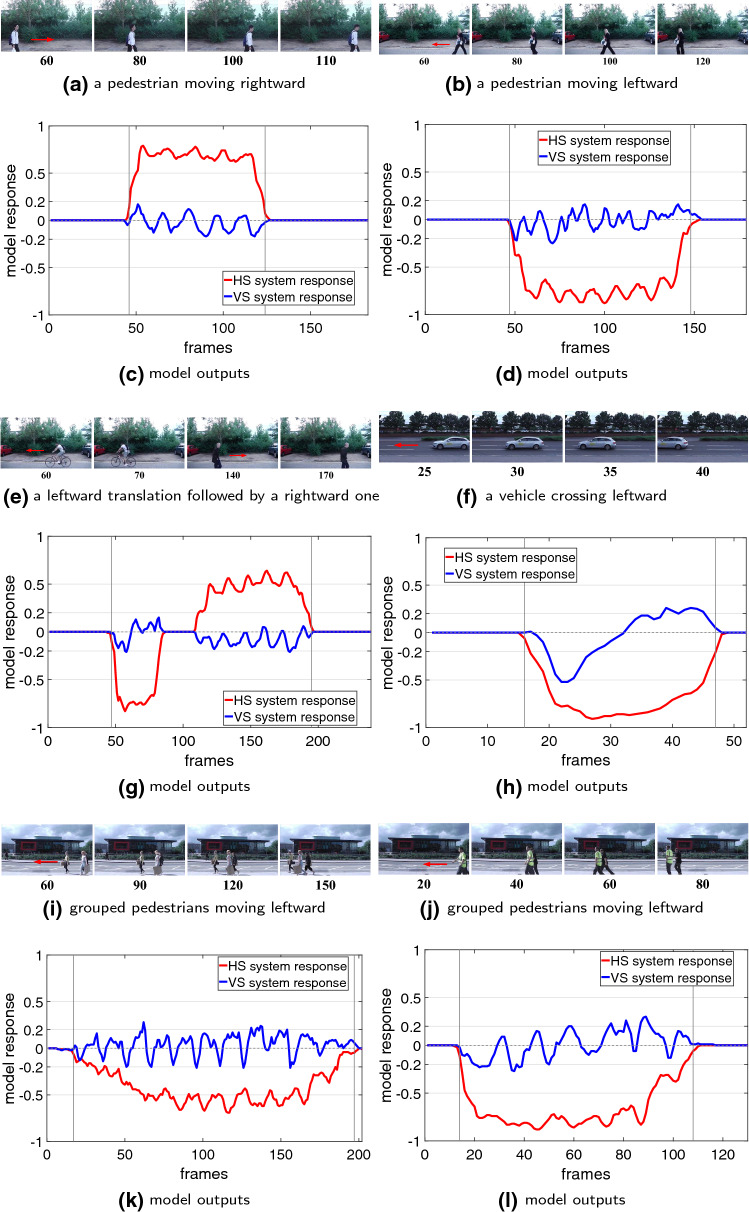
Proposed model responses challenged by foreground translating stimuli in real physical scenes. The frame number is labelled at the bottom of each snapshot. The red arrow in snapshots denotes the ground truth of primary direction of foreground translational OF. The two vertical lines in each result indicate the time window of the appearance of foreground translational OF

In all the experiments, the proposed visual system model was set up in Visual Studio (Microsoft Corporation). The synthetic visual stimuli were generated by a Python open-source library, i.e. the Vision Egg (Straw 2008). The real-world stimuli were recorded by a camera. Data analysis and visualisations were implemented in MATLAB (The MathWorks, Inc., Natick, MA, USA).

## Results

### Demonstrations of the specific direction selectivity

In this kind of tests, we systematically examine the specific direction selectivity of the proposed *Drosophila* visual system model. Firstly, the model is challenged by a few typical motion patterns with clean backgrounds, which include darker and brighter objects translating in four cardinal directions, approaching and receding. The model responses are shown in Figs. 5 and 6. More specifically, when challenged by translating in four cardinal directions (see Fig. 5), the HS and VS systems are highly activated by horizontal and vertical movements, respectively. In the process of translation, the leading and trailing edges of a darker object bring about OFF and ON contrasts, respectively, while a brighter object leads to the opposite responses. As a result, the model with ON and OFF channels can encode both polarity contrast in separate pathways in order to generate the specific DS responses to four cardinal directions: the HS system is rigorously activated by merely the horizontal translational OF representing positive or negative response to its PD (rightward) or ND (leftward) motion; the VS system only responds to the vertical translational OF that also shows positive or negative response to its PD (downward) or ND (upward) motion.

On the other hand, when challenged against approaching and receding motion patterns, i.e. movements in depth (see Fig. 6), both the HS and VS systems are rigorously inhibited during every entire process: the ON and OFF contrast by PD and ND motions with contracting and distracting edges are cancelled by each other. The results demonstrate clearly the direction selectivity of proposed visual system model to translation in four cardinal directions.

Next, the model is tested by more challenging real-world scenarios. Compared to the computer-simulated stimuli, the real physical backgrounds are unstructured including motion distractors, such as the windblown vegetation, etc. As the input visual stimuli, all the translating targets have the ground truth of primary direction in horizontal, as illustrated in Fig. 7. Accordingly, the HS system is highly activated when translations appear in the field of vision. Notably, the VS system is also activated compared to the above stimuli in clean backgrounds, caused by irregular locomotion of translating targets in vertical directions and background distractors. Despite that, the HS system responds much more strongly to the visual stimuli, which can well indicate the principal direction of foreground moving objects. The PD or ND motion in horizontal is well decoded as positive or negative membrane potential of the neural system model. The results verify that the proposed model responds more consistently to foreground translating objects rather than irrelevant background flows that is robust to generate the DS and DO responses against more variable backgrounds.

**Fig. 8:**
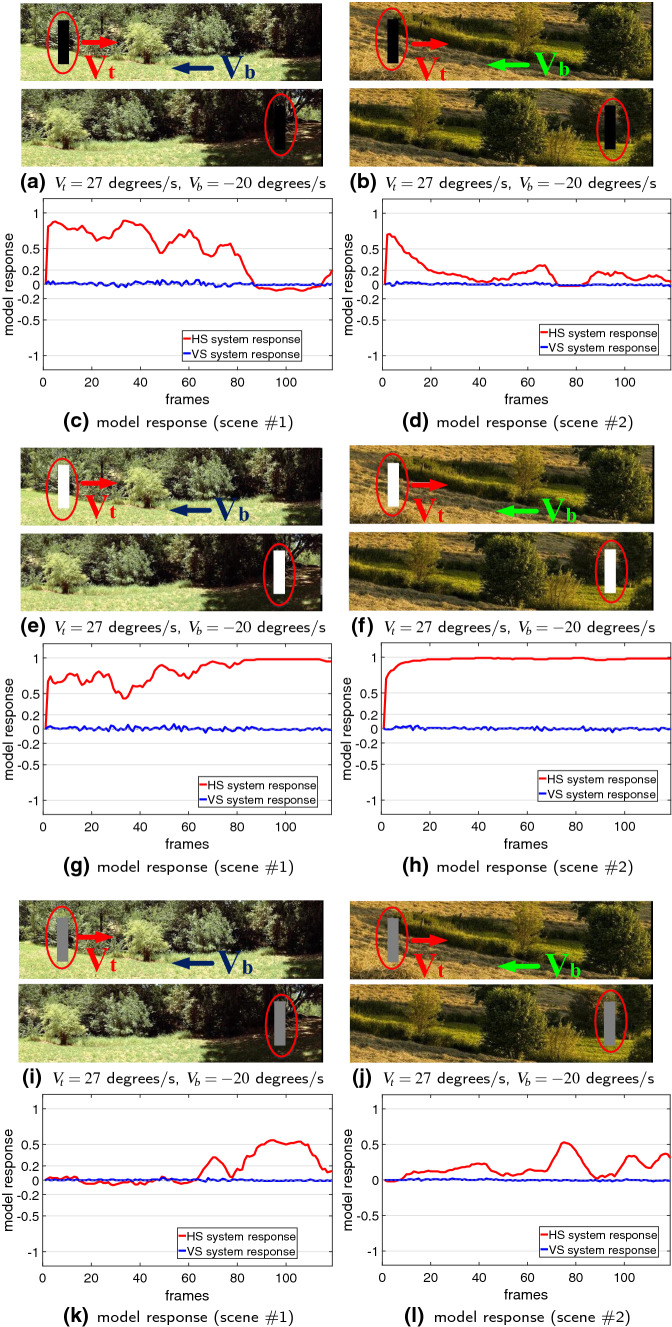
Proposed model responses challenged by two cluttered moving backgrounds. The white, moderate and dark objects start from the left side and translate **rightward** at 27 $^\circ /\mathrm{s}$. The cluttered backgrounds shift **leftward** at $-20 \,^\circ /\mathrm{s}$. The red ellipses mark the start and end positions. The arrows indicate the ground truth directions of foreground objects($V_t$) and moving backgrounds($V_b$)

After that, more systematic experiments are carried on with cluttered moving backgrounds, in which the angular velocities of both the foreground translating objects and the shifting backgrounds are manually controlled. Figure 8 illustrates the model responses by three grey-scale objects moving in front of two shifting natural backgrounds, respectively. We have the following observations:

1. The proposed model is effective to decode the direction of translating objects against cluttered moving backgrounds: only the HS system is activated representing positive response to PD translational motion in front of the ND shifting backgrounds, while the VS system is rigorously suppressed.
2. The model is sensitive to the contrast between translating objects and backgrounds: the white object, with relatively larger contrast to the moving backgrounds, leads to more constant and stronger responses, while the moderate object, with relatively smaller contrast to the moving backgrounds, brings about weaker responses. Moreover, the model is not responding to the dark or moderate object translating in front of the background with little contrast, e.g. the dark object moving into the shadowed area.

**Fig. 9:**
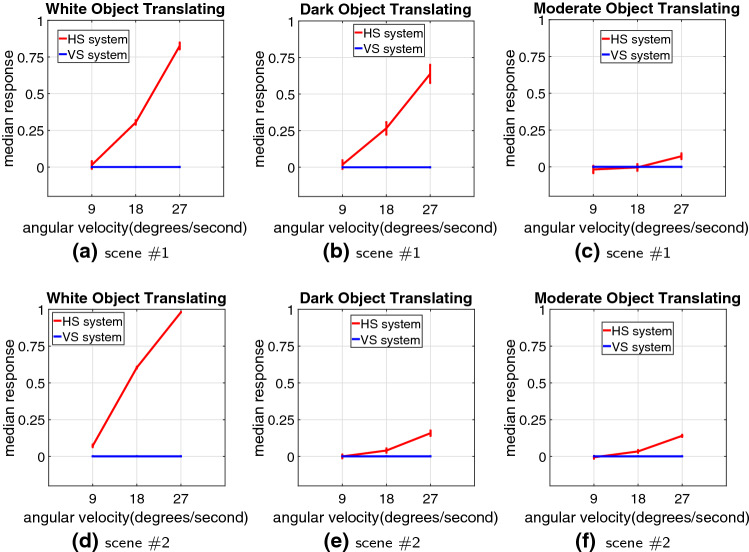
Statistical results of the median responses of HS and VS systems with variance and mean information, challenged by the three grey-scale objects translating at three individual angular velocities, in front of the two moving backgrounds each shifting at a range of angular velocities ($-5, -10, -20,$ $-30, -40\, ^\circ /\mathrm{s}$)

**Fig. 10:**
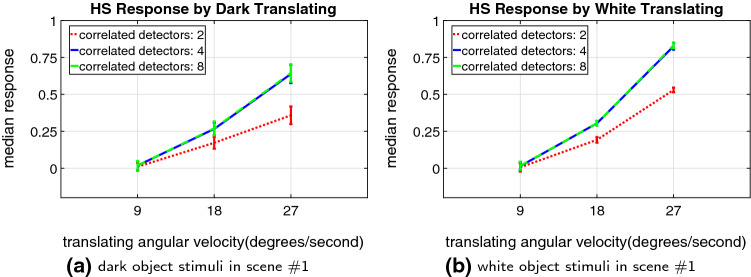
Results of investigation on the different number of correlated motion detectors inside the ON and OFF channels, under the same stimuli settings in Fig. 9

Subsequently, we compare the dynamic response in speed tuning between the three grey-scale translating objects, against the two cluttered moving backgrounds. Based on the experimental setting introduced in Sect. 4, a range of angular velocities for both the foreground targets and the backgrounds are investigated. Figure 9 illustrates the statistical results. Intuitively, the HS system responds more strongly to the PD translating stimuli at faster speeds; while the VS system maintains inactive in all the tests. Importantly, challenged by the ND moving backgrounds at a range of angular velocities, little variance is shown at all tested foreground translation speeds and contrasts, which indicates the proposed model performs robustly and consistently to decode the direction of translation in front of cluttered moving backgrounds. The irrelevant background translational OF mixed with distractors, such as the woods, have been satisfactorily suppressed, which is a significant achievement of this modelling research. Moreover, the model represents a broader dynamic range on the larger-contrast moving target which matches the above observations in Fig. 8.

**Fig. 11:**
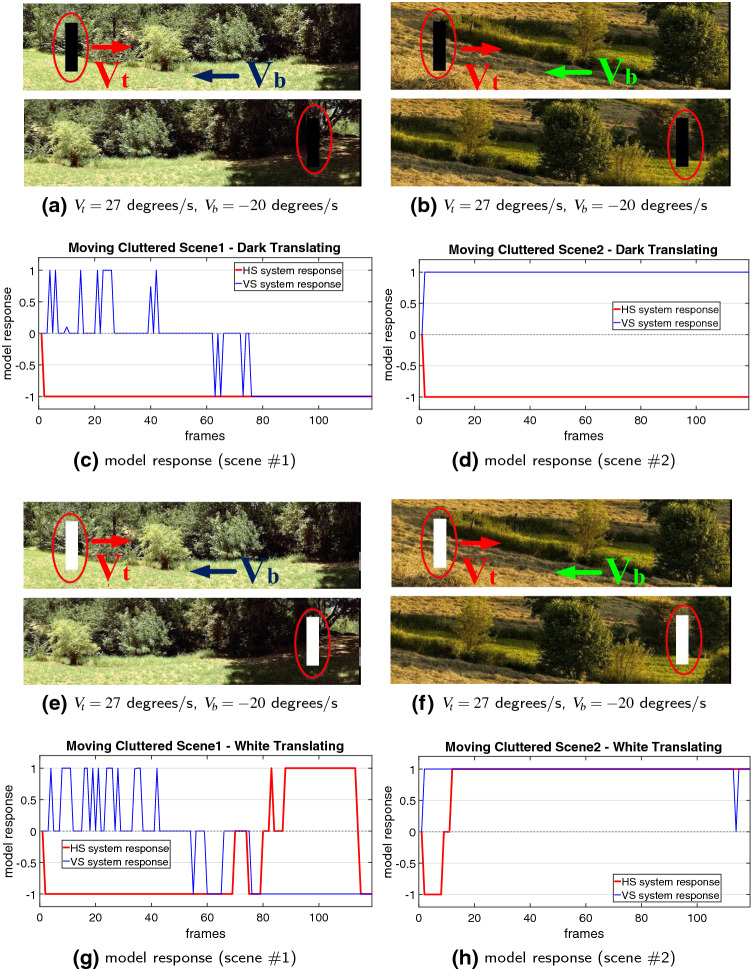
Proposed model responses without the combination of motion pre-filtering mechanisms including the vDoG and the FDSR, challenged by the two cluttered moving backgrounds. The stimuli settings are in accordance with Fig. 8

### Investigations on model characteristics

To provide insight into the significance of proposed new mechanisms or structures in decoding the direction of translating objects against cluttered moving backgrounds, we investigate the effectiveness of spatiotemporal dynamics in the proposed visual system model. Firstly, Fig. 10 demonstrates the effects of ensembles of ON–ON/OFF–OFF local motion correlators on the dynamic response in speed tuning ($n_c$ in Eqs. 11 and 13). The statistical results show that the dynamic response is stable and reflected by all the tested parameter and stimuli settings. The model is expected to respond more strongly to faster moving stimuli with more correlated detectors inside the ON and OFF channels. There is nevertheless little difference between $n_c = 4$ and $n_c = 8$, which indicates that $n_c = 4$ could be an optimal parameter set-up in our case. Such a structure can enhance the dynamic response by alleviating the impact by temporal frequency of visual stimuli though increasing the computational complexity.

**Fig. 12:**
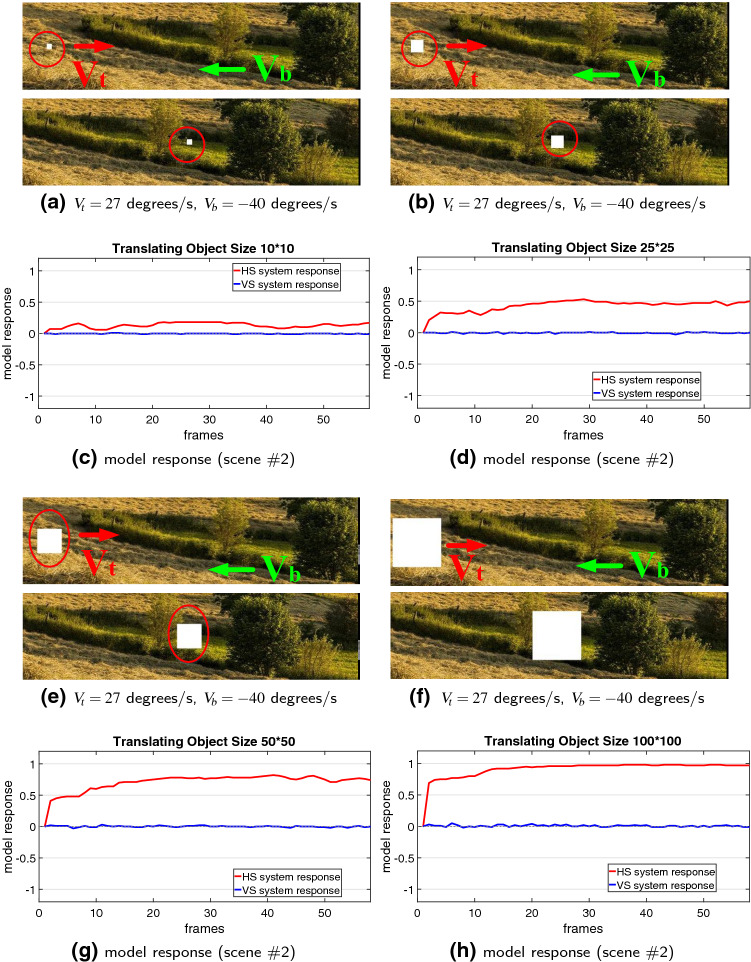
Proposed model responses by different sized white squares translating **rightward** at $27 \,^\circ /\mathrm{s}$, in front of the cluttered background (scene #2) moving **leftward** at $-40 \,^\circ /\mathrm{s}$

Secondly, we compare the performance of model in the absence of proposed combination of spatial (vDoG) and temporal (FDSR) mechanisms refining ON and OFF contrast before generating the DS and DO responses. Figure 11 illustrates the outputs in comparison with Fig. 8. Obviously, without the coordination of proposed spatiotemporal mechanisms, the model is no longer capable of accurately decoding the direction of translating objects in front of the cluttered moving backgrounds. More concretely, the HS system represents negative responses to the ND translational OF caused by the backgrounds, and the VS system is also highly activated. The results further verify the importance of proposed spatiotemporal dynamics to fit with the desired robustness in cluttered moving backgrounds.

**Fig. 13:**
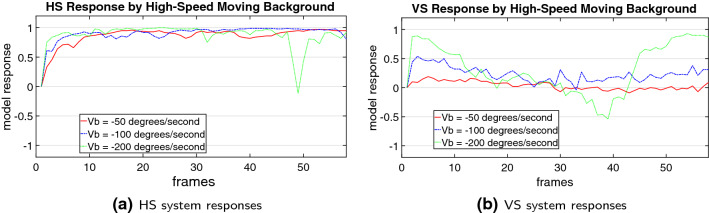
Proposed model responses by the white object ($25 \times 120$ pixels in size) translating rightward at $27 \,^\circ /\mathrm{s}$, embedded in the high-speed and leftward moving background (scene #2) at $-50, -100, -200 \,^\circ /\mathrm{s}$, respectively

Lastly, we also investigate the model performance on visual stimuli possessing different properties including varying sizes of translating objects and higher-speed moving backgrounds. Figures 12 and 13 illustrate the results. Interestingly, the proposed model can detect the different sized targets moving in front of a cluttered, and fast-moving background. However, the model demonstrates responsive preference to larger over smaller sized targets representing stronger response of the HS system, while the VS system is rigorously suppressed by all the tested visual stimuli, as expected (see Fig. 12).

When tested by the very high-speed moving cluttered background, some negative results are obtained: the HS system of the proposed model also responds correctly to the PD motion of foreground translation; the VS system nevertheless is activated more constantly than the afore-tested background angular velocities, especially at the highest velocity of $-200 \,^\circ /\mathrm{s}$ (see Fig. 13). Therefore, the very high-speed cluttered moving background still poses a problem on decoding the direction of foreground moving object.

## Discussion

### Characterisation of the model

We have demonstrated the effectiveness of the proposed *Drosophila* motion vision pathways model for decoding the direction of translating objects in front of different visual backgrounds, from simple to more challenging cluttered moving ones. The visual system model articulates the signal processing behind the OF level, and satisfactorily reproduces the DS and DO responses revealed in the neural circuits. The direction of foreground translating objects is indicated by the global membrane potential of the wide-field HS and VS systems, with which the positive or negative response indicate the PD or ND motion (Figs. 5, 7 and 8). Importantly, the model shows robust direction selectivity and dynamic response to translating objects in front of cluttered moving backgrounds, at a range of tested angular velocities for both the foreground targets and the backgrounds (Figs. 9 and 10). In addition, the model also shows responsive preference to faster-moving (Figs. 9 and 10), larger-size (Fig. 12), higher-contrast (Figs. 8 and 9) translating targets. Moreover, we have clarified the importance of proposed modelling of spatiotemporal dynamics to refine the ON and OFF contrasts prior to the generation of DS responses (Fig. 11), and to improve dynamic response in speed tuning (Fig. 10). Furthermore, we have also shown the existing limitation of the proposed visual system model for foreground translation perception, that is, the model could not suppress the very high-speed background translational OF, to an acceptable level (Fig. 13). In fact, a single-type neural-pathway computation may be insufficient to handle this challenge, whereas the coordination of multiple neural pathways could be a potential solution.

Considering further improvements on this work, we also have the following observations:

1. A recent physiological study has suggested the visual interneurons in the medulla layer of fly motion vision pathways can rapidly adjust the contrast sensitivity (Drews et al. 2020). Therefore, the implementation of contrast normalisation in the proposed model could reduce the high contrast fluctuations in natural images.
2. For the experimental setting, our model processes signals at only 30 Hz that is 8 times lower than the fly’s eye at about 250 Hz, although our model works with 126,000 pixels, 15 times over the fly with approximately 8400 pixels in total. We will further investigate the proposed method by matching the settings with the fly visual systems and applying binocular vision.

### Coordination of multiple neural systems

The experiments have also demonstrated the proposed model can perceive small translating object, and decode its direction under a same model setting. However, in contrast to the STMD models (Wiederman et al. 2008, 2013), the wide-field HS and VS systems have responsive preference to larger-size targets resulting in much stronger responses (Fig. 12). A fascinating future work could be the integration of multiple neural pathways for size-varying target pursuit in natural environments.

Furthermore, the proposed visual system model perfectly complements the looming sensitive neural models like the LGMD (Fu et al. 2018b, 2019a), on the aspect of direction selectivity (Fig. 6). The coordination of them could facilitate the perception of different motion patterns in more challenging scenarios.

## Conclusion

This paper presents computational modelling of the *Drosophila* motion vision pathways accounting for how the flies decode the direction of a moving target in front of highly variable backgrounds. The emphasis herein is laid behind the OF level: the proposed model mimics the visual processing from the photoreceptors, through the parallel ON and OFF pathways, to the LPTCs in four stratified sub-layers sensitive to motion in four cardinal directions. The wide-field HS and VS systems integrate the DS and DO responses from the LPTCs as the model outputs, with which the positive or negative response indicates the PD (rightward, downward) or ND (leftward, upward) translational motion. To extract merely the foreground translation and improve the dynamic response in a cluttered moving background, the proposed modelling of spatiotemporal dynamics including the coordination of motion pre-filtering mechanisms and the ensembles of local correlators inside the ON and OFF channels, works effectively. The experiments have verified the effectiveness of the proposed model with robust direction selectivity in various backgrounds, and also demonstrated its specific responsive preference. The proposed model processes signals in a feed-forward manner resembling the *Drosophila* physiology; its computational efficiency and flexibility could fit with building neuromorphic sensors, either featuring compact size or achieving higher processing speed, for utility in mobile intelligent machines.
